# Role of the *Mycoplasma bovis deoC* gene in nucleoside catabolism and host cell survival

**DOI:** 10.1128/aem.00156-26

**Published:** 2026-05-12

**Authors:** Shijie Geng, Sheik Nadeem Elahee Doomun, Jordi Hondrogiannis, David P. De Souza, Anna Kanci Condello, Glenn F. Browning, Sara M. Klose, Kelly A. Tivendale, Nadeeka K. Wawegama

**Affiliations:** 1 Asia-Pacific Centre for Animal Health, Melbourne Veterinary School, Faculty of Science, The University of Melbourne 188674 , Parkville, Victoria, Australia; 2 Metabolomics Australia, Bio21 Institute of Molecular Science and Biotechnology, The University of Melbourne 195117, Parkville, Victoria, Australia; Washington University in St Louis 7548, St. Louis, Missouri, USA

**Keywords:** *Mycoplasma bovis*, metabolomics, metabolic function, nucleoside catabolism

## Abstract

A putative *deoC* gene (MBOVPG45_0300) is critical for the survival of the important bovine pathogen *Mycoplasma bovis* in co-culture with Madin-Darby bovine kidney (MDBK) cells. To investigate the function of this gene, combined metabolomic analyses were performed to compare the polar metabolite profiles of a mutant strain with a transposon insertion into the MBOVPG45_0300 gene (∆MBOVPG45_0300) with those of the parent strain PG45. Steady-state metabolomic analysis showed that the ∆MBOVPG45_0300 mutant had significantly lower abundances of metabolites in nucleoside metabolism and significantly higher abundances of intermediates in the pentose phosphate pathway. An isotope labeling study using ^13^C_5_-thymidine revealed that deoxyribonucleoside catabolism was disrupted in ∆MBOVPG45_0300 and that the production of acetyl-coenzyme A was affected. Metabolomic footprinting suggested that the disruption of MBOVPG45_0300 resulted in a compensatory increase in uptake of pyruvate. These different metabolomic analyses of the metabolic changes associated with disruption of MBOVPG45_0300 suggested that this gene played a critical role in the catabolism of deoxyribonucleosides in *M. bovis*.

**IMPORTANCE:**

Mycoplasmas are reliant on host cells for the acquisition of nutrients, and their metabolic functions are vital for both survival and virulence. Metabolomic analysis can be used to determine the metabolic functions of genes in mycoplasmas by examining the metabolic changes in mutants with gene disruptions. We combined different metabolomic techniques to study the metabolic changes in the important bovine pathogen *Mycoplasma bovis* caused by the disruption of the putative *deoC* gene, which is essential for the survival of this organism in association with host cells. Our results suggest the product of the gene plays a role in the catabolism of deoxyribonucleosides and that this metabolic pathway is critically important in the interactions of *M. bovis* with host cells.

## INTRODUCTION

Mycoplasmas are a group of bacteria in the class *Mollicutes* that evolved from the gram-positive bacteria (1). Many are pathogens that cause diseases in humans, animals, insects, or plants (2–5). The reductive evolution of these organisms has resulted in the loss of many biosynthetic capabilities, resulting in a dependence on the acquisition of many complex nutrients from the host for both survival and virulence (6, 7). Although the functions of the products of several genes that play roles in the survival and virulence of the important bovine pathogen *Mycoplasma bovis* have been described (7, 8), the functions of the products of many genes and their role in the pathogenesis of diseases caused by this pathogen still remain to be fully elucidated.

Metabolomic analyses have been used in several studies to examine the metabolic functions of genes in mycoplasmas. Comparative profiling of the metabolomes of transposon mutants and their parental strain has enabled the identification of the substrates of several mycoplasma transport proteins (9–11). However, metabolomic profiling is limited in its ability to detect dynamic changes in metabolites and pathways (12). Stable-isotope labeling studies, which enable the shunting of the labeled substrate into downstream pathways to be traced, have been used to study metabolic pathways of mycoplasmas in more detail (13). Compensatory substrate transport has been detected in some mutants (9, 10), suggesting the need to integrate extracellular metabolomic profiling to obtain more comprehensive information about metabolism in mycoplasmas.

Our previous studies examining the capacity of different mutants of *M. bovis* to proliferate in co-culture with Madin-Darby bovine kidney (MDBK) cells found that a putative deoxyribose-5-phosphate aldolase (DERA) gene, *deoC* (MBOVPG45_0300), is essential for the survival of *M. bovis* in association with host cells (14). In the study described here, intracellular and extracellular metabolomic profiling and stable-isotope labeling studies were performed to compare the *M. bovis* parent strain PG45 and the transposon mutant with a disruption of the *deoC* gene with the aim of detecting the metabolic changes in the transposon mutant and determining the function of the product of the *deoC* gene.

## MATERIALS AND METHODS

### Bioinformatic analysis

Annotations of genes were retrieved from the UniProt and BioCyc databases (15). The Conserved Domains database (CDD) from NCBI was used to identify conserved domains in the protein sequence, while protein topology was predicted using the online topology prediction server DeepTMHMM (https://dtu.biolib.com/DeepTMHMM). Operon structures in *M. pneumoniae* M129 were obtained from a previous study (16), while predicted operons in other mycoplasma species were obtained from the BioCyc database. Homologs of proteins in other mycoplasma species were identified using BlastP to search the non-redundant protein sequence database (nr) from the National Centre for Biotechnology Information (NCBI) (https://www.ncbi.nlm.nih.gov).

### Mycoplasma strains and culture conditions

The stock culture of *M. bovis* parent strain PG45, hereafter referred to as PG45, was obtained from the Asia-Pacific Centre for Animal Health (APCAH) culture collection at The University of Melbourne, Victoria, Australia. The PG45 stock culture was diluted 1:10 in *M. bovis* growth medium (0.6% heart infusion broth, 1% protease peptone no.3, 0.5% NaCl, 3.7% yeast extract, 10% inactivated swine serum, 0.0064% phenol red, and 300 μg penicillin/mL, with the pH adjusted to 7.8) and incubated at 37°C for 24 h to obtain working cultures for the following experiments. The viable titer of the PG45 working cultures was determined to be 9.77 × 10^8^ color changing units (CCU)/mL using a titration method described previously (10). The *M. bovis* mutant strain, hereafter referred to as ∆MBOVPG45_0300, was generated in a previous study by transposon mutagenesis (17) and contains a Tn*4001* transposon insertion in the MBOVPG45_0300 locus of the genome (transposon insertion point between nucleotide positions 336,603 and 336,604). Working cultures of ∆MBOVPG45_0300 were prepared as described above for the PG45 strain, with the addition of 50 μg gentamicin sulfate/mL to the *M. bovis* growth medium to maintain selective pressure for the presence of the transposon. The viable titer of the ∆MBOVPG45_0300 working cultures was determined to be 6.13 × 10^8^ CCU/mL. To confirm the presence of the transposon in ∆MBOVPG45_0300, PCR amplification using a primer complementary to MBOVPG45_0300 (5′-CTAGTAGCCGCCGTGTGATT-3′) and an inverse primer complementary to the IR of the transposon (5′-TGGCCTTTTTACTTTTACACAAT-3′) was performed as previously described (10), and PCR products were visualized by agarose gel electrophoresis. Sanger sequencing was used to determine the sequence of the PCR product, and it was aligned with the reference genome sequence of *M. bovis* PG45 (GenBank accession number CP002188) to confirm the location of the transposon in MBOVPG45_0300.

### Growth curves of PG45 and ∆MBOVPG45_0300 in *M. bovis* growth medium

Growth curves were generated to determine the mid-logarithmic phase of growth for both *M. bovis* strains. Working cultures of PG45 and ∆MBOVPG45_0300 were diluted 1:40 in *M. bovis* growth medium and incubated at 37°C for up to 18 h. Viable titers were determined for both strains at 0 h and then every 2 h from 10 to 18 h after inoculation.

### Steady-state metabolomic analysis of intracellular metabolites using gas chromatography-mass spectrometry (GC-MS)

Five biological replicates of PG45 and six biological replicates of ∆MBOVPG45_0300 were prepared by diluting working cultures 1:40 in 10 mL of *M. bovis* growth medium and incubating them at 37°C until they reached the mid-logarithmic phase of growth. Metabolic quenching was performed by infusing 9.5 mL of mid-logarithmic phase cultures with 28.5 mL of ice-cold 1× phosphate-buffered saline (PBS), followed by a 5-min incubation in an ice-water slurry. To minimize the degradation of metabolites during sampling, mycoplasma cells were harvested by centrifugation at 2°C (20,000 × *g*, 20 min), and washed twice with ice-cold 1× PBS at 2°C (17,000 × *g*, 5 min). Methods for metabolite extraction and derivatization were adapted from a previous study (13). To extract intracellular metabolites, mycoplasma cell pellets were resuspended in 250 μL of a chloroform:methanol:H_2_O (1:3:1) mixture containing 1 μM of each internal standard (^13^C_6_-sorbitol and ^13^C_5_^15^N-valine). After agitation (4°C, 10 min) and centrifugation (16,048 × *g*, 4°C, 10 min), a CHRIST RVC 2-33 CD plus speed vacuum concentrator was used to evaporate a 100 μL sample at 30°C. The dried samples were derivatized by mixing them with 25 μL of methoxyamine hydrochloride (30 mg/mL pyridine, Merck) and shaking the mixture at 37°C for 2 h, then adding 25 μL of *N, O-bis* (trimethylsilyl) trifluoracetamide with trimethylchlorosilane and incubating the mixture at 37°C for 1 h. For GC-MS detection, 1 μL of each derivatized sample was injected onto an Agilent DB-5 column in a 2030 Shimadzu gas chromatograph coupled with a TQ8050NX triple quadrupole mass spectrometer (Shimadzu, Japan). Metabolites were identified by comparing the retention time and fragmented ion patterns to those of the endogenous metabolites in the Shimadzu Smart Metabolite Database, and data matrices with relative peak areas of each metabolite were generated for further data processing.

### Stable isotope labeling with ^13^C_3_-lactate and ^13^C_5_-thymidine

The methods used for stable isotope labeling of *M. bovis* were adapted from a previous study (13). Three biological replicates of *M. bovis* PG45 and ∆MBOVPG45_0300 were prepared by diluting working cultures 1:40 in 10 mL of *M. bovis* growth medium supplemented ^13^C_3_-lactate at 5 mM or ^13^C_5_-thymidine at 28 μM, and then incubating them until the mid-logarithmic phase of growth was reached. Metabolic quenching, extraction, and GC-MS detection of metabolites for the ^13^C_3_-lactate labeling study were performed as described above for steady-state metabolomics. Liquid chromatography (LC)-MS detection was used for the ^13^C_5_-thymidine labeling study because of its enhanced sensitivity for the detection of nucleoside metabolites. The metabolic quenching procedure was the same as that used for the GC-MS platform, but different metabolic extraction and detection methods were used. Mycoplasma cell pellets were resuspended in 250 μL of a chilled chloroform:methanol:H_2_O mixture (1:3:1) containing 2 μM ^13^C_5_^15^N-valine and 2 μM leucine-d_3_ as internal standards. Samples were vortexed and incubated with continuous agitation in a Thermomixer C (Eppendorf) at 4°C for 10 min, then centrifuged at 16,048 × *g*, 4°C for 10 min. A 225 µL volume of supernatant was transferred into high-performance liquid chromatography (HPLC) inserts and evaporated under nitrogen using the MICROVAP Nitrogen Evaporation System. The dried extracts were resuspended in 75 µL of an acetonitrile:water mixture (4:1), and metabolite detection was performed on a Vanquish Horizon UHPLC system (Thermo Scientific) coupled to an Orbitrap ID-X Tribrid mass spectrometer (Thermo Scientific). Metabolites were identified using TraceFinder (Thermo Scientific) and El-Maven (https://www.elucidata.io/el-maven), based on matching accurate mass and retention time to the 550 authentic standards in the Metabolomics Australia in‐house library.

### Metabolomic footprinting analysis of extracellular metabolites using GC-MS

Metabolomic footprinting analysis was performed as described previously, with some minor changes (13). In brief, three biological replicates of *M. bovis* PG45 and ∆MBOVPG45_0300 were prepared by diluting working cultures 1:40 in 100 mL of *M. bovis* growth medium and incubating them at 37°C until they reached the mid-logarithmic phase of growth. The cultures were then centrifuged (18,700 × *g*, 20 min, 2°C) to pellet the mycoplasma cells, which were then resuspended in 1 mL of fresh *M. bovis* growth medium and incubated at 37°C for up to 60 min. A 30 μL sample was taken at 0, 15, 30, and 60 min. Samples were immediately quenched in a dry ice-ethanol bath followed by centrifugation at 17,000 × *g* at 0°C for 10 min. Extraction of metabolites from the supernatant and GC-MS detection were performed as described above.

### Colony size measurement

*M. bovis* PG45 and ∆MBOVPG45_0300 working stocks were diluted 1:40 in *M. bovis* growth medium and incubated at 37°C for 18 h. The cultures were then diluted 1:100,000 in pre-warmed fresh *M. bovis* growth medium, and inoculated onto pre-warmed sheep blood agar plates, which were then incubated at 37°C for 9 days. Images of *M. bovis* colonies were captured using a SZX12 Stereo Microscope (Olympus) and the DinoXcope software (Version 2.4.1), and the diameters of 20 colonies of each strain were measured using ImageJ (Version 2.14.0/1.54f).

### Cryoelectron microscopy of *M. bovis*

*M. bovis* PG45 and ∆MBOVPG45_0300 working stocks were diluted 1:40 in 30 mL of fresh *M. bovis* growth medium and incubated at 37°C until the culture reached the mid-logarithmic phase of growth. Mycoplasma cells were harvested by centrifugation (20,000 × *g*, 2°C, 20 min), and washed twice with ice-cold 1× PBS (17,000 × *g*, 2°C, 5 min). The cells were resuspended in 200 μL of ice-cold 1× PBS and deposited onto a Lacey carbon-coated 200 mesh copper grid, then plunge frozen in liquid ethane using a Vitrobot Mark IV (Thermofisher), and examined using a Titan Krios cryoelectron microscope (300 kV; −20 μm defocus). Micrographs were taken with a Gatan K3 direct detector (29.77 electrons per nm^2^), and the cross-sectional areas (in μm^2^) of 213 PG45 cells and 121 ∆MBOVPG45_0300 cells were determined using ImageJ (Version 2.14.0/1.54f).

### Statistical analysis

Statistical analysis of the metabolomics data matrix generated from GC-MS was performed using MetaboAnalyst (https://www.metaboanalyst.ca). The data matrix was normalized by median and log transformed, followed by principal component analysis (PCA) and partial least squares discriminant analysis (PLS-DA), and *t*-tests incorporating the Benjamini and Hochberg false discovery rate (FDR) approach were used to identify the metabolites with significant differences in abundance. In order to identify changes in the abundance of metabolites between strains, the fold change (FC) in abundance of each metabolite in the steady-state metabolomic analysis was calculated by dividing the average median-normalized peak area in ∆MBOVPG45_0300 by that of the parent strain. Metabolites with an FC < 0.77 and an FDR-adjusted *P* value < 0.05 were considered to be significantly less abundant in the mutant compared to the PG45 parent strain, while metabolites with an FC > 1.30 and an FDR-adjusted *P* value < 0.05 were considered to be significantly more abundant in the mutant. Pathway enrichment analysis for the steady-state metabolomic data matrix was performed using MetaboAnalyst, based on metabolic pathways from the Kyoto Encyclopedia of Genes and Genomes (KEGG) database. To determine the changes in metabolites over time in the metabolomic footprinting study, the FC of each metabolite was calculated by dividing the median-normalized peak area at 15 min, 30 min, and 60 min by that at 0 min. The peak area data of metabolites in the isotope labeling study were normalized to the internal standards and the cell count to compare their abundance in each strain. The mean ± standard deviation (SD) of the colony diameters and cross-sectional areas of the cells of each strain were calculated. The cross-sectional areas of mycoplasma cells were square root transformed, and the significance of differences between the two strains in the sizes of colonies and cells was determined using unpaired *t*-tests in GraphPad Prism (Version 9.4.1), with a *P* value < 0.05 considered significant.

## RESULTS

### Bioinformatic analysis suggests MBOVPG45_0300 is a putative *deoC* gene

**Fig 1:**
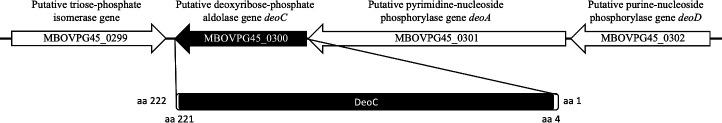
Gene context of MBOVPG45_0300 with annotations shown above each gene locus and the DeoC family domain identified in MBOVPG45_0300. aa, amino acid.

Both BioCyc and UniProt databases annotated MBOVPG45_0300 as a *deoC* gene, encoding DERA, an enzyme catalyzing the reversible conversion between deoxyribose-5-phosphate (dR5P) and glyceraldehyde-3-phosphate (GAP). A BlastP search found that MBOPVG45_0300 was homologous to the DERA protein (MHO_3330) of *Mycoplasma hominis* (18), with 58.77% identity (*E* value = 8 × 10^−86^). CDD identified a DERA (or DeoC family) domain between residues 4 and 221 in MBOVPG45_0300 (*E* value = 3.07 × 10^−117^) (Fig. 1). Gene context analysis using the BioCyc database showed that MBOVPG45_0300 was located in an operon with a putative pyrimidine nucleoside phosphorylase gene, *deoA* (MBOVPG45_0301), and a putative purine nucleoside phosphorylase gene, *deoD* (MBOVPG45_0302), located immediately upstream (Fig. 1). In addition, a putative triose-phosphate isomerase gene (MBOVPG45_0299) was located in the inverse orientation immediately downstream of MBOVPG45_0300 (Fig. 1). The protein topology predicted by DeepTMHMM indicated that MBOVPG45_0300 was an intracellular protein without transmembrane structures (Fig. S1).

### Steady-state metabolomic profiling suggests the disruption in MBOVPG45_0300 altered nucleoside metabolism and the pentose phosphate pathway

**Fig 2:**
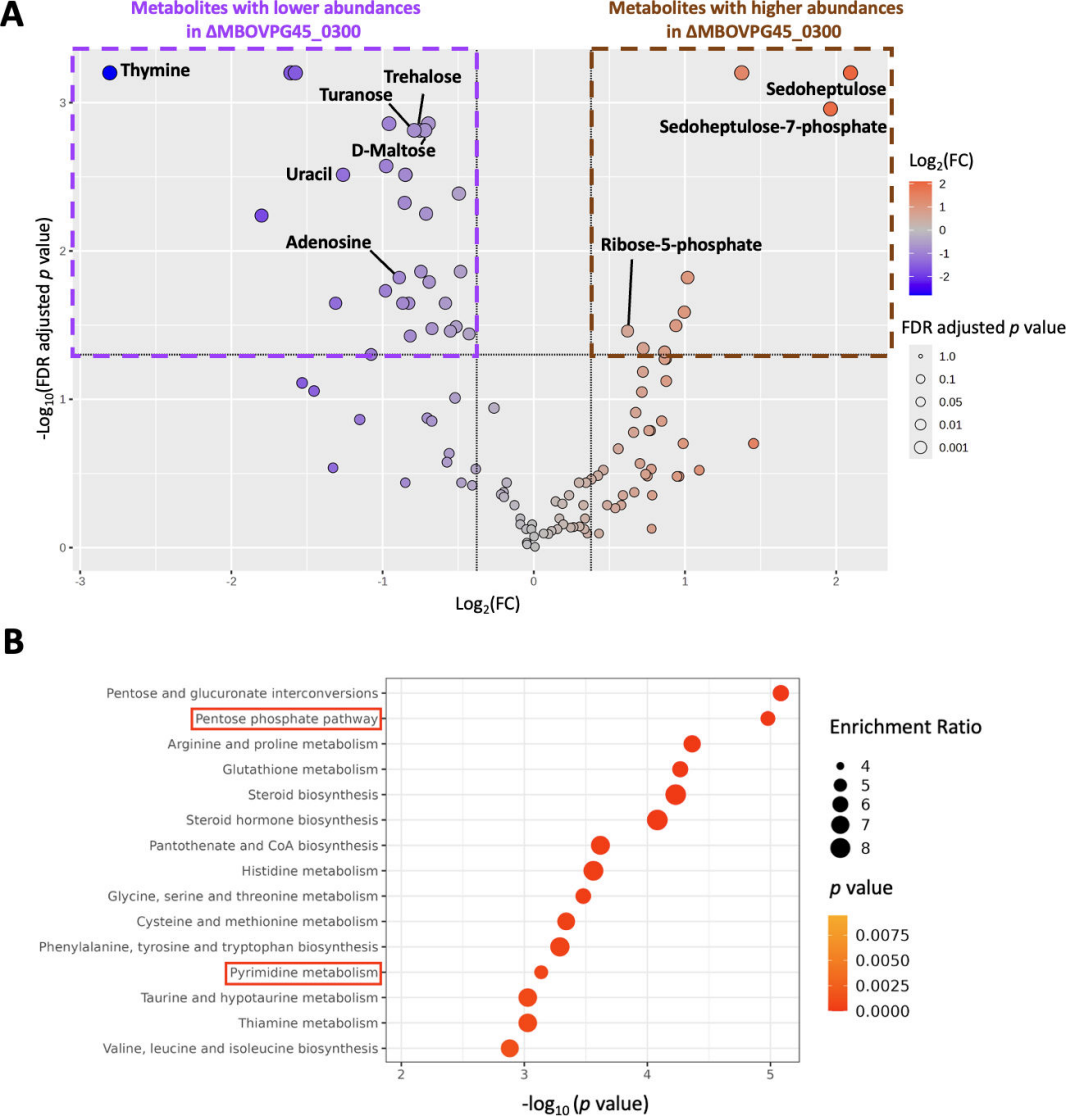
Steady-state metabolomic profiling for PG45 and ∆MBOVPG45_0300. (**A**) Metabolites with significantly higher or lower abundances in ∆MBOVPG45_0300 compared to PG45. Purple dots above the horizontal dashed line and to the left of the left vertical dashed line indicate metabolites with significantly lower abundances (fold change [FC] < 0.77, FDR-adjusted *P* value < 0.05) in the mutant, while red dots above the horizontal dashed line and to the right of the right vertical dashed line indicate metabolites with significantly higher abundances (FC > 1.30, FDR-adjusted *P* value < 0.05) in the mutant. (**B**) The 15 pathways significantly enriched with metabolites with differential abundances (*P* value < 0.05) between PG45 and ∆MBOVPG45_0300 with the lowest *P* values. Each dot represents an independent pathway in KEGG. The different sizes and color intensities of the dots indicate different enrichment ratios and *P* values, respectively.

Both PG45 and ∆MBOVPG45_0300 had reached the mid-logarithmic phase of growth at 14 h after inoculation, with a viable titer of 8.48 × 10^8^ CCU/mL (Fig. S2), when the mycoplasma cells were collected and analyzed by GC-MS (Table S1). PCA and PLS-DA detected separate clusters for PG45 and ∆MBOVPG45_0300 (Fig. S3). The abundances of 40 metabolites differed significantly between the groups (Table S2), with significantly higher abundances of 9 metabolites (FC > 1.30, FDR-adjusted *P* value < 0.05) and significantly lower abundances of 31 metabolites (FC < 0.77, FDR-adjusted *P* value < 0.05) in ∆MBOVPG45_0300 compared to PG45 (Fig. 2A).

Three metabolites in nucleoside metabolism had significantly lower abundances in ∆MBOVPG45_0300, including thymine, uracil, and adenosine, while thymine had the lowest fold change between the two strains (approximately 0.14 times the relative abundance in the parent strain) (Fig. 2A). Thymidine was the only deoxyribonucleoside detected by GC-MS, and its abundance did not differ significantly between the strains (Table S1). Although there were no significant differences between the strains in the abundances of detectable glycolysis intermediates (3-phosphoglyceric acid [3PG], 2-phosphoglyceric acid [2PG], phosphoenolpyruvic acid [PEP], pyruvate, and lactate) (Table S1), three intermediates in the pentose phosphate pathway were significantly more abundant in ∆MBOVPG45_0300 than in PG45 (ribose-5-phosphate [R5P], sedoheptulose, and sedoheptulose-7-phosphate) (Fig. 2A). In addition, disaccharides, including turanose, trehalose, and maltose, had significantly lower abundances in ∆MBOVPG45_0300 compared with PG45 (Fig. 2A). Pathway enrichment analysis found that the pentose phosphate pathway and pyrimidine metabolism differed significantly between PG45 and ∆MBOVPG45_0300 (Fig. 2B).

### Stable-isotope labeling demonstrated that catabolism of deoxyribonucleosides was disrupted in ∆MBOVPG45_0300

**Fig 3:**
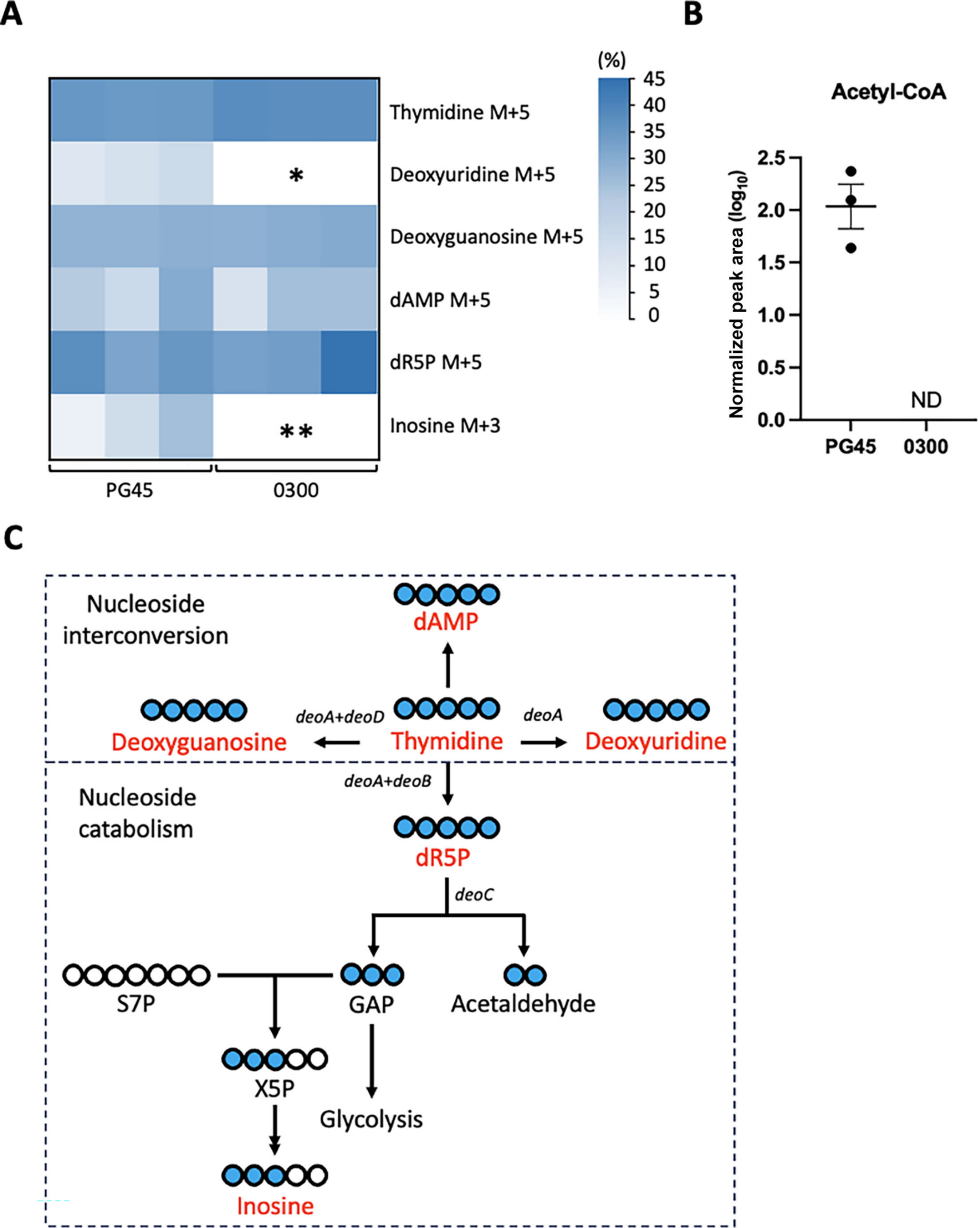
Stable-isotope labeling of metabolites after supplementation with ^13^C_5_-thymidine. (**A**) Proportions of M + 5 or M + 3 isotopologues in the total pools of metabolites detectable in *M. bovis* PG45 (PG45) and ∆MBOVPG45_0300 (0300). (**B**) Comparison of the abundances of acetyl-coenzyme A in the two strains. (**C**) Utilization of labeled carbons in the interconversion and catabolism of thymidine in *M. bovis* PG45 and the role of the *deo* genes in the associated pathways. Labeled metabolites are shown in red, and metabolic reactions are indicated with arrows and labeled with the name(s) of the gene(s) responsible for the conversion. Blue circles above the nucleosides indicate the number of labeled carbons in the sugar moiety. Blue and white circles in the other metabolites indicate the number of labeled and unlabeled carbons in the compounds, respectively. The metabolic map was adapted from a previous study (13). Acetyl-CoA, acetyl coenzyme A; dAMP, deoxyadenosine monophosphate; dR5P, deoxyribose-5-phosphate; GAP, glyceraldehyde-3-phosphate; ND, not detected; S7P, sedoheptulose-7-phosphate; X5P, xylulose-5-phosphate. *P < 0.05 and **P < 0.01.

The function of MBOVPG45_0300 was further studied by performing stable-isotope labeling studies with ^13^C_3_-lactate and ^13^C_5_-thymidine, and peak areas of isotopologues in the stable isotope labeling study are shown in Tables S3 and S4. Labeled nucleoside metabolites were not identified in mycoplasma cells cultured in growth medium supplemented with ^13^C_3_-lactate (Table S5). Six metabolites in the nucleoside metabolic pathway were isotopically labeled in PG45 after supplementation of the growth medium with ^13^C_5_-thymidine (Fig. 3A and C). These metabolites included one nucleotide (deoxyadenosine monophosphate [dAMP] M + 5), three nucleosides (thymidine M + 5, deoxyuridine M + 5, and deoxyguanosine M + 5), dR5P M + 5, and inosine M + 3 (Fig. 3A and C). There were significantly lower proportions of the M + 5 isotopologue in the total pool of deoxyuridine and of the M + 3 isotopologue in the total pool of inosine in ∆MBOVPG45_0300 compared to PG45, with no deoxyuridine M + 5 or inosine M + 3 detected in the mutant strain (Fig. 3A). No significant difference was detected between the strains in the proportions of labeled isotopologues in the total pools of the other four metabolites (Fig. 3A). Acetyl-coenzyme A (acetyl-CoA) in the glycolytic pathway, which was detected in PG45, was undetectable in ∆MBOVPG45_0300 (Fig. 3B).

### Metabolomic footprinting suggests that uptake of ribonucleosides was not affected and that uptake of pyruvate was upregulated in ∆MBOVPG45_0300

**Fig 4:**
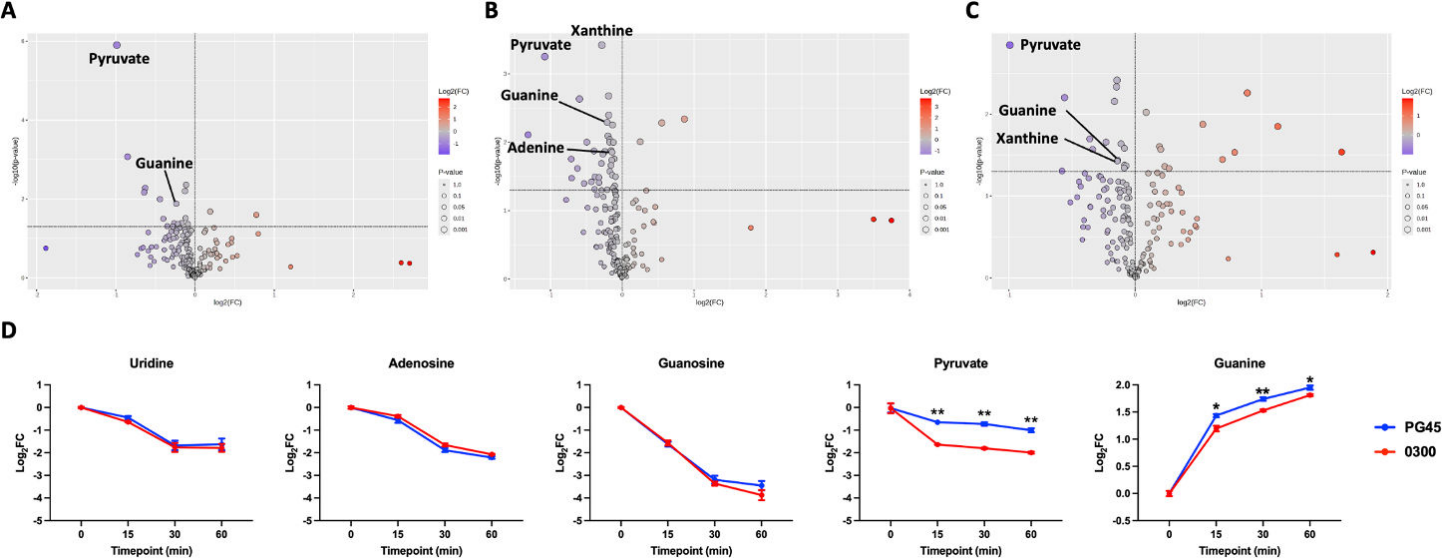
Metabolomic footprinting analysis of ∆MBOVPG45_0300 and PG45 showing supernatant components with significantly different fold changes after (**A**) 15 min, (**B**) 30 min, and (**C**) 60 min of incubation. Purple dots above the dashed horizontal line indicate components with significantly lower fold changes in the supernatant of ∆MBOVPG45_0300 compared to that of PG45 (*P* value < 0.05), while red dots indicate components with significantly higher fold changes (*P* value < 0.05). Medium components associated with nucleoside metabolism or pyruvate metabolism are identified. (**D**) Log_2_FC values of supernatant components at different time points; *P < 0.05 in the multiple comparisons of PG45 and ∆MBOVPG45_0300 at the same time point; **P < 0.01 in the multiple comparisons of PG45 and ∆MBOVPG45_0300 at the same time point.

Three nucleosides (uridine, adenosine, and guanosine), one nucleobase (cytosine), four organic acids (pyruvate, lactate, 2-hydroxybutyric acid, and oxoglutaric acid), and one sterol (cholesterol) had significantly lower abundances in the culture supernatant of PG45 over time (*P* value < 0.05), while nucleobases, sugars, organic acids, and amino acids had significantly higher abundances in the culture supernatant of PG45 over time (*P* value < 0.05) (Tables S6 and S8). Significantly lower fold changes in multiple medium components were detected in the culture supernatant of ∆MBOVPG45_0300 compared to that of PG45—16 after 15 min of incubation (Fig. 4A), 37 after 30 min of incubation (Fig. 4B), and 17 after 60 min of incubation (Fig. 4C). Some medium components had significantly higher fold changes in the culture supernatant of ∆MBOVPG45_0300 compared to that of PG45—2 after 15 min of incubation (Fig. 4A), 3 after 30 min of incubation (Fig. 4B), and 11 after 60 min of incubation (Fig. 4C). The rates of depletion of uridine, adenosine, and guanosine from the culture supernatant did not differ significantly between ∆MBOVPG45_0300 and PG45 (Fig. 4D). Pyruvate had significantly faster rates of depletion in the culture supernatant of ∆MBOVPG45_0300 compared to that of PG45 over the entire incubation period (Fig. 4D). In addition, guanine had significantly slower rates of accumulation in the culture supernatant of ∆MBOVPG45_0300 over the entire incubation period (Fig. 4D), while adenine had significantly slower rates of accumulation in the culture supernatant of ∆MBOVPG45_0300 at 30 min (Fig. 4B), and xanthine had significantly slower rates of accumulation at 30 and 60 min (Fig. 4B and C).

### Homologs of *M. bovis* nucleoside metabolism-associated proteins in other mycoplasma species

**Fig 5:**
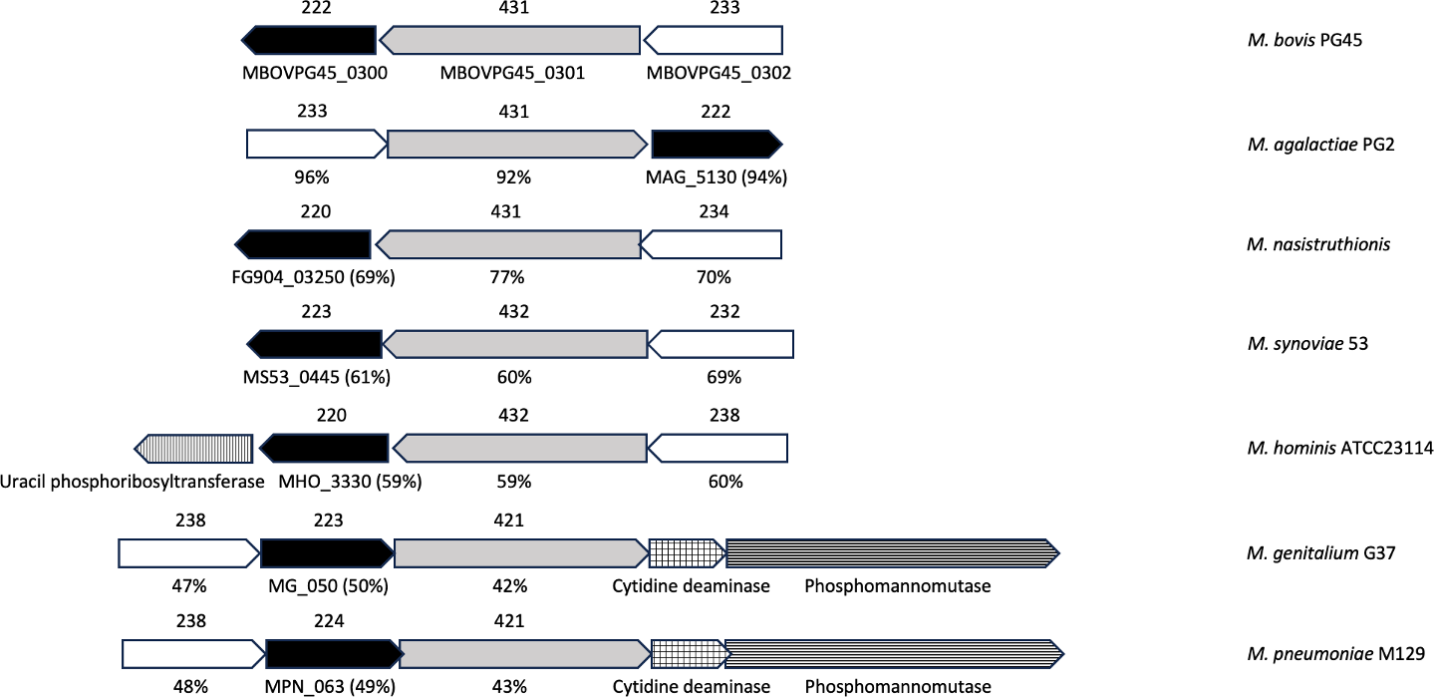
Operon analysis of homologs of MBOVPG45_0300, MBOVPG45_0301, and MBOVPG45_0302 in different mycoplasma species. Homologs are shown as arrows with the same color and pattern. The numbers above and percentage values below the arrows indicate the protein size (number of amino acids) and the amino acid sequence identity with the *M. bovis* PG45 homolog, respectively. Additional genes in the operons are labeled with the predicted functions of their products.

Homologs of MBOVPG45_0300, MBOVPG45_0301, and MBOVPG45_0302 were identified in a number of mycoplasma species (Fig. 5). The homologs of MBOVPG45_0301 and MBOVPG45_0302 were located immediately upstream of the MBOVPG45_0300 homologs in *M. agalactiae* strain PG2, *M. nasistruthionis*, *M. synoviae* strain 53, and *M. hominis* strain ATCC23114 (Fig. 5). In *M. genitalium* strain G37 and *M. pneumoniae* strain M129, the homologs of MBOVPG45_0302 were located immediately upstream of MBOVPG45_0300 and the homologs of MBOVPG45_0301 were located immediately downstream of MBOVPG45_0300 (Fig. 5). In addition, a putative uracil phosphoribosyltransferase gene was located immediately downstream of the MBOVPG45_0300 homolog in the operon in *M. hominis* strain ATCC23114, while a putative cytidine deaminase gene and a putative phosphomannomutase gene were located immediately downstream of the MBOVPG45_0301 homolog in *M. genitalium* strain G37 and *M. pneumoniae* M129 operons (Fig. 5).

### Colonies of ∆MBOVPG45_0300 were smaller than those of PG45, while the cells of ∆MBOVPG45_0300 were larger than those of PG45

**Fig 6:**
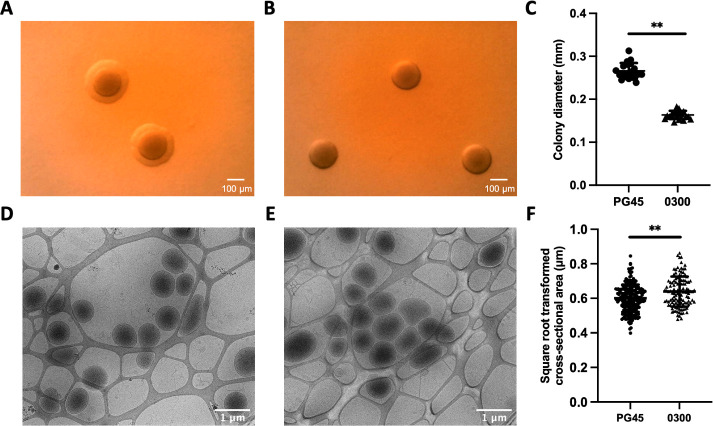
Light micrographs of representative colonies of (**A**) PG45 and (**B**) ∆MBOVPG45_0300. (**C**) Plots of the diameters of colonies of PG45 and ∆MBOVPG45_0300 (0300). Cryo-electron micrographs of (**D**) PG45 cells and (**E**) ∆MBOVPG45_0300 cells. (**F**) Plots of cross-sectional areas of PG45 and ∆MBOVPG45_0300 (0300) cells after square root transformation. In panels C and F, the horizontal lines indicate the mean; **P value < 0.01.

Light photomicrographs of representative colonies of PG45 and ∆MBOVPG45_0300 are shown in Fig. 6A and B. The mean colony diameter for ∆MBOVPG45_0300 was 0.16 ± 0.01 mm, which was significantly smaller than that of PG45 (0.27 ± 0.02 mm) (Fig. 6C). Cryo-electron micrographs of PG45 and ∆MBOVPG45_0300 cells are shown in Fig. 6D and E. The mean of the square root transformed cross-sectional area of ∆MBOVPG45_0300 cells was 0.64 ± 0.09 µm, while that of PG45 cells was 0.60 ± 0.08 µm (Fig. 6F). The mean square root transformed cross-sectional area of ∆MBOVPG45_0300 cells was significantly greater at the mid-logarithmic phase of growth than that of PG45 cells (Fig. 6F).

## DISCUSSION

**Fig 7:**
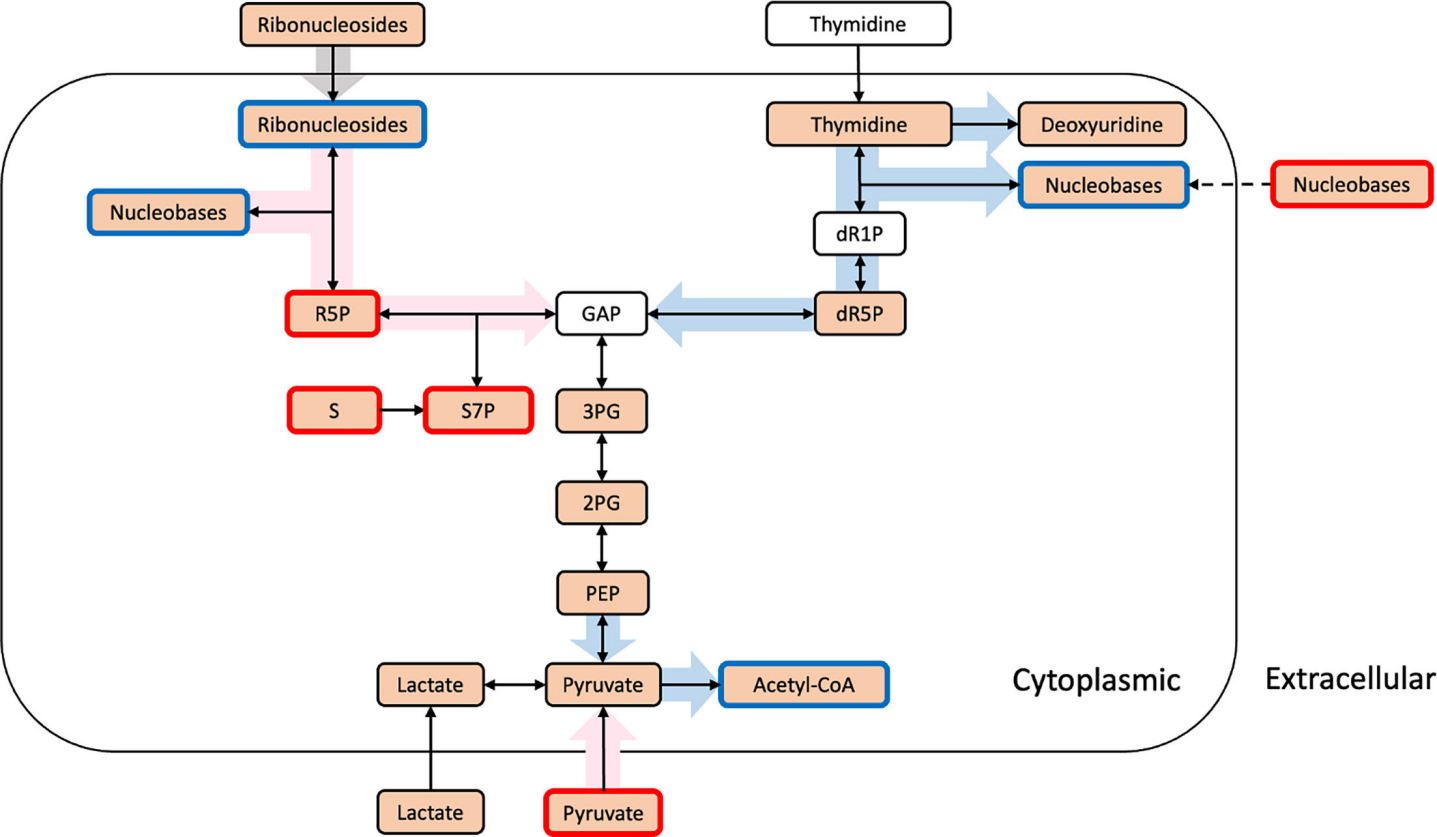
Schematic summary of the metabolic changes in ∆MBOVPG45_0300 detected in these studies. Orange and white shaded rectangles indicate detectable and undetectable metabolites, respectively. A red outline indicates intracellular metabolites with a higher abundance in ∆MBOVPG45_0300 compared to PG45 and medium components with faster rates of depletion from the culture supernatant of ∆MBOVPG45_0300 compared to that of PG45. A blue outline indicates intracellular metabolites with a lower abundance. A black outline indicates intracellular metabolites that did not differ in abundance or medium components that did not differ in their rates of depletion between ∆MBOVPG45_0300 and PG45. Red shaded arrows indicate upregulated metabolic flux, blue shaded arrows indicate downregulated metabolic flux, and the gray arrow indicates unchanged metabolic flux in ∆MBOVPG45_0300 inferred from these studies. 2PG, 2-phosphoglyceric acid; 3PG, 3-phosphoglyceric acid; Acetyl-CoA, acetyl-coenzyme A; dR1P, deoxyribose-1-phosphate; dR5P, deoxyribose-5-phosphate; GAP, glyceraldehyde-3-phosphate; PEP, phosphoenolpyruvic acid; R5P, ribose-5-phosphate; S, sedoheptulose; S7P, sedoheptulose-7-phosphate.

Combined metabolomic profiling was performed in this study to investigate the metabolic function of MBOVPG45_0300, which has been shown previously to be essential for the survival of *M. bovis* in association with host cells. Our findings from different metabolomic approaches enabled a comprehensive analysis of the metabolic consequences of the disruption of MBOVPG45_0300 in *M. bovis*, as shown in Fig. 7. The results from stable-isotope tracing studies suggested that the deoxyribonucleoside catabolic pathway was disrupted in ∆MBOVPG45_0300, consistent with the detection of significantly lower abundances of nucleobases in the mutant by steady-state metabolomic profiling. In addition, metabolomic footprinting suggested that the production of pyruvate from glycolysis was disrupted in ∆MBOVPG45_0300, and that the upregulation of the pentose phosphate pathway in the mutant identified by steady-state metabolomic profiling may have a compensatory role in the maintenance of carbon metabolism in *M. bovis*. Consistent with predictions that MBOVPG45_0300 is a *deoC* gene in the *deoDAC* operon, this study indicated that the metabolic role of this gene is in deoxyribonucleoside catabolism in *M. bovis*.

DERA, encoded by the *deoC* gene, catalyzes the reversible conversion of dR5P to GAP (19), and has been characterized in a number of bacterial species, including *Escherichia coli* (20), *Bacillus cereus* (21), and *Salmonella enterica* (22). The *deoC* gene is one of four genes in the *deo* operon, with the other three being *deoA*, *deoB*, and *deoD* (23). Purine phosphorylase, encoded by *deoD*, and thymidine/pyrimidine phosphorylase, encoded by *deoA,* are responsible for the interconversion of (deoxy)ribonucleosides and (deoxy)ribose-1-phosphate, while phosphopentomutase, encoded by *deoB*, catalyzes the interconversion of (deoxy)ribose-1-phosphate and (deoxy)ribose-5-phosphate, which can enter central carbon metabolism through the pentose phosphate pathway or, after conversion by DERA to GAP, the glycolytic pathway (23). The *deo* operon has been identified as a metabolic link between nucleosides and carbon metabolism, supported by its important role in deoxyribonucleoside catabolism (24, 25) and biosynthesis (26). In mycoplasmas, both ribose and thymidine have been identified as carbon sources, indicating the presence of nucleoside catabolism (18, 27). In addition, a previous study integrating proteomic and indispensable gene data predicted the activity of phosphopentomutase and DERA in nucleotide biosynthesis in both *M. pneumoniae* and *M. agalactiae* (28). In the intracellular metabolomic profiling we have reported here, the abundances of nucleoside metabolites and intermediates in the pentose phosphate pathway were significantly different in ∆MBOVPG45_0300 and PG45 (Fig. 7), suggesting a key metabolic role of MBOVPG45_0300 in nucleoside and carbon metabolism.

Previous isotope tracing studies in *M. bovis*, using ^13^C-labeled glucose, glycerol, and pyruvate, failed to detect the shunt of carbon into the triose part of the glycolytic pathway (13). Lactate, which was found to be significantly depleted in the supernatant of *M. bovis* cultures (13), has been suggested as a potential carbon source for nucleotide biosynthesis through gluconeogenesis in *M. agalactiae*, a species closely related to *M. bovis* (28). In our study, labeling studies with ^13^C_3_-lactate did not detect any labeled intermediate of nucleoside metabolism, suggesting there was no carbon flux from lactate to nucleosides. Addition of DNA to cell culture medium promotes growth of *M. bovis*, suggesting deoxyribonucleosides may act as a carbon source (29), so we also performed labeling studies using ^13^C_5_-thymidine. The detection of deoxyuridine M + 5 and deoxyguanosine M + 5 in labeled cultures of strain PG45 suggested the presence of both purine and pyrimidine phosphorylase activity in *M. bovis*, while the detection of dAMP M+5 indicated that nucleosides were also channeled into nucleotide synthesis. The metabolite dR5P is an important intermediate in deoxyribonucleoside catabolism and can induce the expression of the enzymes encoded in the *deo* operon (30). We detected dR5P M + 5 and inosine M + 3 in ^13^C_5_-thymidine-labeled PG45, suggesting the activity of phosphopentomutase and DERA, which are responsible for the shunting of carbon into the deoxyribonucleoside catabolic pathway. Unlike a previous ^13^C_5_-thymidine labeling study, which detected accumulation of labeled lactate (31), our study failed to detect any labeled glycolytic intermediates. This may be due to the capacity of *M. bovis* to oxidize lactate as an energy source (32), which suggests the shunt of carbon from glycolysis to acetyl-CoA for energy generation, as has been observed in *M. agalactiae* (28). Deoxyuridine M + 5 was not detected in ∆MBOVPG45_0300, suggesting that pyrimidine phosphorylase activity, encoded by *deoA*, was disrupted in the mutant (Fig. 7). The absence of detectable inosine M + 3 in ∆MBOVPG45_0300 also suggested that the shunt of carbon to the catabolic pathway was disrupted in the mutant (Fig. 7). These results were consistent with the prediction that MBOVPG45_0300 was a *deoC* gene and suggested that it played an important role in the deoxyribonucleoside catabolic pathway.

Acetyl-CoA, which has been detected in PG45, was undetectable in ∆MBOVPG45_0300, suggesting either disruption of or a faster metabolic flux in pyruvate metabolism downstream of glycolysis. We also performed extracellular metabolomic profiling to determine the impact of the ∆MBOVPG45_0300 mutation on nutrient uptake in *M. bovis*. The significantly lower or higher abundances of nucleoside metabolites, sugars, organic acids, and amino acids in the culture supernatant of PG45 suggested the depletion or production, respectively, of these components by *M. bovis*, consistent with previous metabolomic footprinting analyses of the organism (13, 33). The significantly faster rate of depletion of pyruvate in the culture supernatant of ∆MBOVPG45_0300 suggested that the uptake of pyruvate was upregulated in the mutant, and that the undetectable acetyl-CoA in the organism was due to reduced metabolic flux from glycolysis (Fig. 7). These results were also consistent with the disruption of the deoxyribonucleoside catabolic activity in the mutant, and the potential regulatory effects of nucleosides on pyruvate metabolism identified in a previous study (29). Additionally, the upregulated pyruvate uptake in ∆MBOVPG45_0300 detected in our study indicates a likely compensatory mechanism to maintain energy generation. DNA, the major source of deoxyribonucleosides, is abundant in tissues and in cell cultures as it is released from living cells and during cellular breakdown (34). In eukaryotic cells, pyruvate plays a vital metabolic role connecting glycolysis and the tricarboxylic acid (TCA) cycle and is used as either an energy source or biosynthetic substrate (35). A previous study has shown that replication of *Mycoplasma felis* is significantly increased in Crandell-Rees feline kidney (CRFK) cells during co-infection with another bacterial pathogen *Chlamydia felis* (*C. felis*), while infection with *C. felis* alone caused a shift to anabolism in CRFK cells (36). This suggests the effect of nutrient accessibility on the replication of mycoplasmas in host cells, and, therefore, the relatively limited access of pyruvate to ∆MBOVPG45_0300 during co-culture with host cells may result in an insufficient supply of carbon and energy, and, eventually, abrogated growth.

R5P, produced by ribonucleoside catabolism, is linked to glycolysis through the pentose phosphate pathway (37, 38). In our study, the absence of changes in ribonucleoside uptake and the higher abundances of intermediates in the pentose phosphate pathway in ∆MBOVPG45_0300 suggested that the significantly lower abundance of ribonucleosides in the mutant was due to upregulated catabolism (Fig. 7). This is also consistent with the reduced metabolic flux in both deoxyribonucleoside catabolism and pyruvate metabolism, suggesting a potential compensatory effect in *M. bovis* to maintain central carbon metabolism. The significantly slower rate of accumulation of nucleobases in the culture supernatant of ∆MBOVPG45_0300 suggested the upregulation of an alternative strategy for the maintenance of nucleobase recycling in the mutant, and that the lower abundance of intracellular nucleobases might be due to the disruption of deoxyribonucleoside phosphorylase activity (Fig. 7).

A previous metabolomic study suggested that *M. bovis* could import glucose and disaccharides, as evidenced by the intracellular accumulation of these sugars, and glucose was identified as a carbon source for the salvage pathway of purines and pyrimidines (13). The significantly lower abundances of disaccharides (turanose, trehalose, and maltose) in ∆MBOVPG45_0300 compared to PG45 suggest that the utilization of these sugars for the biosynthesis of nucleoside metabolites may have been upregulated in the mutant.

As alterations in carbon metabolism can affect cell growth through the control of cell cycle progression in bacteria (39), we further explored whether there were any morphological differences between PG45 and ∆MBOVPG45_0300. In *Bacillus subtilis*, pyruvate maintains normal cell division by affecting the function of the cell division protein FtsZ, and cells with division defects are longer and have a filamentous morphology (40). In mycoplasmas, pyruvate metabolism has been shown to affect the growth rate of *M. agalactiae*, with disruption of the pyruvate dehydrogenase complex resulting in smaller colony sizes (41). In addition, the abundance of FtsZ was found to be greater in proteomic analyses of *M. hominis* grown in a medium with thymidine as the carbon source instead of arginine (42), while disruption of glycerol metabolism in *M. gallisepticum* affects its cell size. Consistent with these previous studies, ∆MBOVPG45_0300 produced smaller colonies and had larger cells than PG45, suggesting a potential impact of disrupted carbon metabolism on the growth rate and cell division of *M. bovis*. Although beyond the scope of this study, further transcriptomic or proteomic analyses may help determine any potential effects of MBOVPG45_0300 on the expression of genes and proteins involved in growth and division of *M. bovis*.

In conclusion, the combined metabolomic profiling studies described here suggested that the deoxyribonucleoside catabolic pathway and pyruvate metabolism were disrupted, and that the ribonucleoside catabolic pathway was upregulated, in ∆MBOVPG45_0300 compared to PG45, consistent with the bioinformatic annotation of MBOVPG45_0300 as a *deoC* gene. These comprehensive analyses of the metabolism of ∆MBOVPG45_0300, which had abrogated growth in association with host cells, emphasized the importance of nucleoside availability in the interactions of *M. bovis* with its host and demonstrated that interference with pathways for import of nucleosides could attenuate virulence in this mycoplasma species, enabling development of novel control strategies for the disease it causes.

## Data Availability

Raw data matrices generated by GC-MS and LC-MS in steady-state metabolomic and isotope labeling studies are provided as supplemental material (Tables S1, S3, and S4).

## SUPPLEMENTAL MATERIAL

Figures S1 to S3 aem.00156-26-s0001.pdf Topology of the protein product of MBOVPG45_0300 predicted by DeepTMHMM, growth curves of *M. bovis* PG45 and ΔMBOVPG45_0300 (0300) in *M. bovis* growth medium, and differences in the intracellular metabolomic profiles of *M. bovis* PG45 and ΔMBOVPG45_0300.

Table S1 aem.00156-26-s0002.xlsx Raw data matrix generated by GC-MS in steady-state metabolomic analysis.

Table S2 aem.00156-26-s0003.xlsx Significantly different metabolites in ΔMBOVPG45_0300 compared with *M. bovis* PG45.

Table S3 aem.00156-26-s0004.xlsx Peak areas of isotopologues in the stable isotope labeling study with labeled lactate.

Table S4 aem.00156-26-s0005.xlsx Peak areas of isotopologues in the stable isotope labeling study with labeled thymidine.

Tables S5 to S8 aem.00156-26-s0006.docx Proportions of labeled carbon in the metabolites detected in the ^13^C_3_-lactate labeling experiment; supernatant components in metabolomic footprinting studies of PG45 with significant fold changes in abundance.

ASM does not own the copyrights to Supplemental Material that may be linked to, or accessed through, an article. The authors have granted ASM a non-exclusive, world-wide license to publish the Supplemental Material files. Please contact the corresponding author directly for reuse.
