# Supplementary material for: Role of the Mycoplasma bovis deoC gene in nucleoside catabolism and host cell survival
Source: Appl Environ Microbiol. 2026 May 12;92(6):e00156-26. doi: 10.1128/aem.00156-26 (PMC13274396; doi:10.1128/aem.00156-26)
Supplement: Figures S1 to S3 — Topology of the protein product of MBOVPG45_0300 predicted by DeepTMHMM, growth curves of M. bovis PG45 and ΔMBOVPG45_0300 (0300) in M. bovis growth medium, and differences in the intracellular metabolomic profiles of M. bovis PG45 and ΔMBOVPG45_0300. [file aem.00156-26-s0001.pdf]

**Supplementary figures**

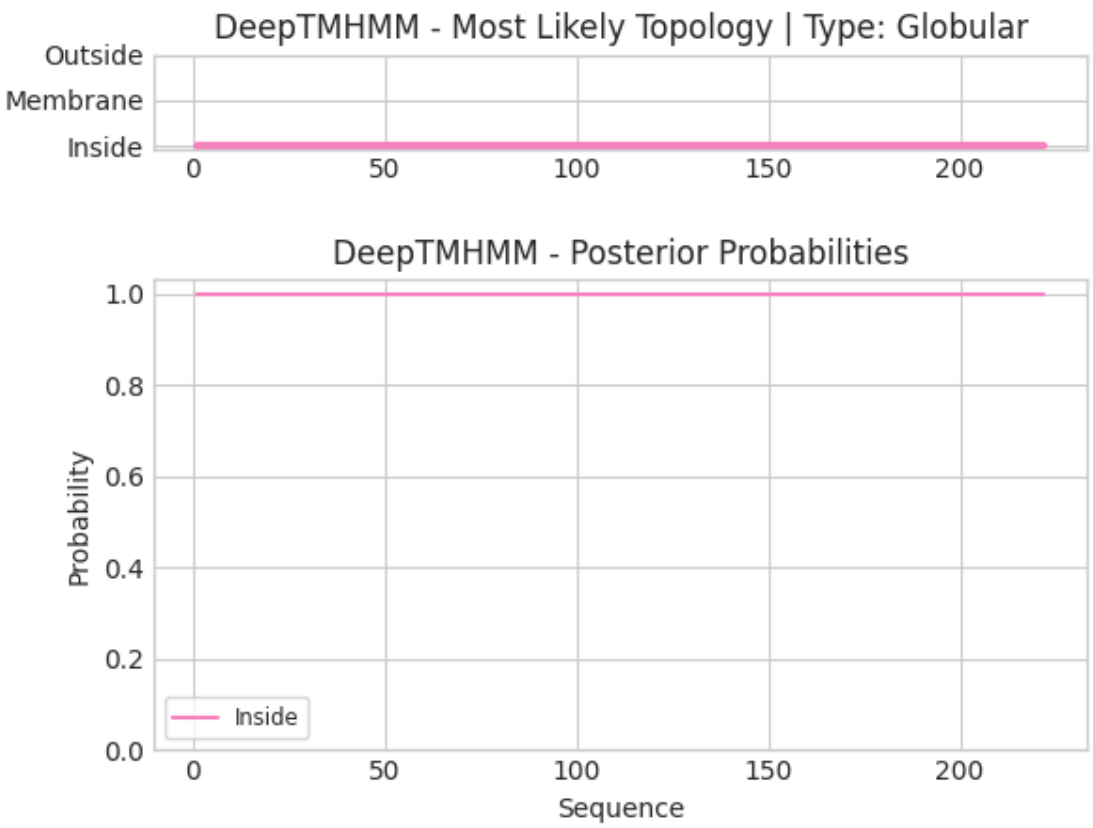


**Figure S1.** Topology of the protein product of MBOVPG45_0300 predicted by DeepTMHMM.

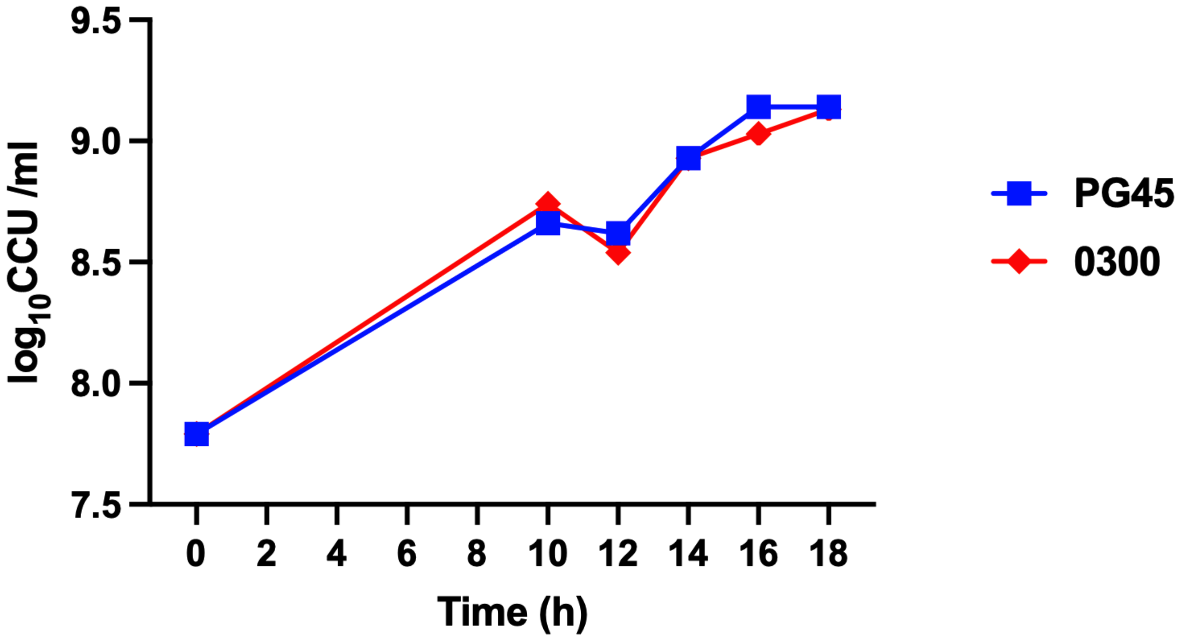


**Figure S2.** Growth curves of *M. bovis* PG45 and ΔMBOVPG45_0300 (0300) in *M. bovis* growth medium.

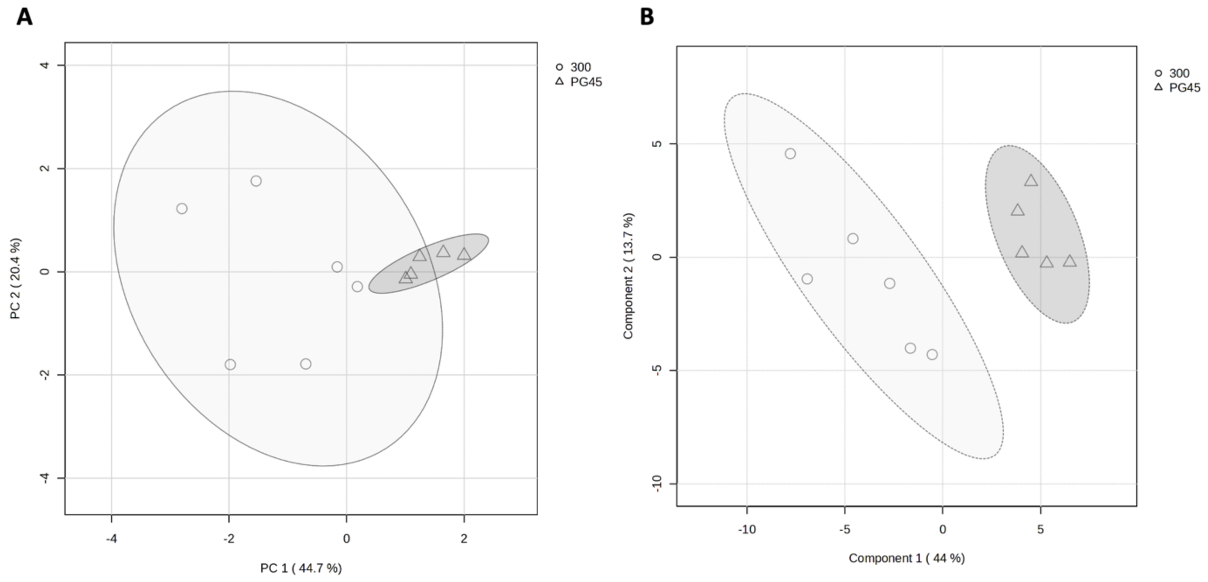


**Figure S3.** Differences in the intracellular metabolomic profiles of *M. bovis* PG45 and ΔMBOVPG45_0300 (300) identified by (A) principal component analysis and (B) partial least squares discriminant analysis, with the 95% confidence regions indicated by the shaded ovals.
