# Supplementary material for: Role of the Mycoplasma bovis deoC gene in nucleoside catabolism and host cell survival
Source: Appl Environ Microbiol. 2026 May 12;92(6):e00156-26. doi: 10.1128/aem.00156-26 (PMC13274396; doi:10.1128/aem.00156-26)
Supplement: Tables S5 to S8 — Proportions of labeled carbon in the metabolites detected in the 13C3-lactate labeling experiment; supernatant components in metabolomic footprinting studies of PG45 with significant fold changes in abundance. [file aem.00156-26-s0006.docx]

| **Table S5. Proportions (%) of labelled carbon in the metabolites detected in the ^13^C_3_-lactate labelling experiment.** | | | | | | |
| --- | --- | --- | --- | --- | --- | --- |
| **Compounds** | **PG45_1** | **PG45_2** | **PG45_3** | **0300_1** | **0300_2** | **0300_3** |
| Lactate | 5.04 | 4.12 | 3.37 | 5.53 | 5.4 | 5.46 |
| Alanine | 1.85 | 1.92 | 2.37 | 2.87 | 2.53 | 3.05 |
| Proline | 6.81 | 6.84 | 7.32 | 12.03 | 10.64 | 11.05 |
| Aspartate | 1.68 | 1.74 | 1.73 | 2.48 | 2.93 | 2.48 |
| 5-oxoproline | 1.4 | 1.41 | 1.4 | 2.19 | 2.36 | 2.43 |
| Glycerol-3-phosphate | 1.2 | 1.23 | 1.28 | 2.03 | 2.4 | 1.79 |
| Glucose | 1.22 | 1.21 | 1.43 | 2.28 | 2.37 | 2.14 |
| Palmitic acid | 7.44 | 7.34 | 7.29 | 7.36 | 7.29 | 7.26 |
| Stearic acid | 8.88 | 9.02 | 8.92 | 8.94 | 8.77 | 8.97 |
| Trehalose | 1.43 | 1.4 | 1.55 | 2.64 | 2.71 | 2.19 |
| Cholesterol | 1.6 | 1.67 | 1.69 | 3.04 | 2.9 | 2.71 |
| Serine | 1.08 | 1.04 | 1.24 | 1.97 | 1.5 | 2.49 |

| **Table S6. Supernatant components in metabolomic footprinting studies of PG45 with significant fold changes (FC) in abundance at 15 min compared to 0 min.** | | | | |
| --- | --- | --- | --- | --- |
| **Compound** | **FC** | **log_2_(FC)** | **Raw *p* value** | **-log_10_(*p*)** |
| 2-deoxy-D-ribose | 2.4809 | 1.3108 | 0.0092752 | 2.0327 |
| 3-hydroxyisovaleric acid | 1.1115 | 0.1525 | 0.022395 | 1.6499 |
| 3-phenyllactic acid | 1.6255 | 0.70087 | 0.00121 | 2.9172 |
| 3-sulfinoalanine | 1.2621 | 0.33581 | 0.040189 | 1.3959 |
| 4-hydroxyproline | 1.1309 | 0.17752 | 0.025042 | 1.6013 |
| 5-aminopentanoic acid | 1.2457 | 0.31691 | 0.0027018 | 2.5683 |
| Adenine | 1.8543 | 0.89084 | 1.07E-05 | 4.9692 |
| Adenosine | 0.67989 | -0.55663 | 0.0076005 | 2.1192 |
| Aspartic acid | 1.0807 | 0.11193 | 0.014612 | 1.8353 |
| Cholesterol | 0.90157 | -0.14949 | 0.045869 | 1.3385 |
| Glutamic acid | 1.0971 | 0.13371 | 0.00080824 | 3.0925 |
| Glyceric acid | 1.1036 | 0.14218 | 0.042814 | 1.3684 |
| Glycerol-3-phosphate | 1.0995 | 0.13687 | 0.012318 | 1.9095 |
| Guanine | 2.7019 | 1.434 | 5.96E-06 | 5.2247 |
| Guanosine | 0.32674 | -1.6138 | 0.00021785 | 3.6618 |
| Hydrocinnamic acid | 1.8361 | 0.87666 | 0.0099537 | 2.002 |
| Hypoxanthine | 1.64 | 0.71366 | 0.00010111 | 3.9952 |
| Isoleucine | 1.0935 | 0.129 | 0.03283 | 1.4837 |
| Mannitol | 1.1802 | 0.23909 | 0.045395 | 1.343 |
| Methionine | 1.0893 | 0.12335 | 0.0050057 | 2.3005 |
| Phenylalanine | 1.0906 | 0.12514 | 0.012995 | 1.8862 |
| Pyroglutamic acid | 1.0881 | 0.12175 | 0.04074 | 1.39 |
| Pyruvic acid | 0.63701 | -0.65062 | 0.035402 | 1.451 |
| Serine | 1.1139 | 0.15557 | 0.0022676 | 2.6444 |
| Uracil | 1.7332 | 0.7934 | 0.0018736 | 2.7273 |
| Uridine | 0.73418 | -0.44579 | 0.014615 | 1.8352 |

| **Table S7. Supernatant components in metabolomic footprinting studies of PG45 with significant fold changes (FC) in abundance at 30 min compared to 0 min.** | | | | |
| --- | --- | --- | --- | --- |
| **Compound** | **FC** | **log_2_(FC)** | **Raw *p* value** | **-log_10_(*p*)** |
| 2-deoxy-D-ribose | 4.4809 | 2.1638 | 9.26E-06 | 5.0334 |
| 2-hydroxybutyric acid | 0.87106 | -0.19916 | 0.014301 | 1.8446 |
| 3-phenyllactic acid | 1.699 | 0.76468 | 0.012753 | 1.8944 |
| 4-hydroxyproline | 1.1604 | 0.21464 | 0.0011715 | 2.9312 |
| Adenine | 2.3722 | 1.2462 | 4.17E-06 | 5.3802 |
| Adenosine | 0.27149 | -1.881 | 2.56E-05 | 4.5916 |
| Alanine | 1.0486 | 0.068401 | 0.0012291 | 2.9104 |
| Arginine | 1.2071 | 0.27156 | 0.02706 | 1.5677 |
| Asparagine | 1.1163 | 0.15873 | 0.0096164 | 2.017 |
| Aspartic acid | 1.0704 | 0.098169 | 0.0028735 | 2.5416 |
| Citramalic acid | 1.4453 | 0.53138 | 0.024108 | 1.6178 |
| Glutamic acid | 1.1175 | 0.16023 | 1.82E-05 | 4.7393 |
| Glutathione | 1.1395 | 0.1884 | 0.011715 | 1.9313 |
| Glyceric acid | 1.1039 | 0.14263 | 0.0063459 | 2.1975 |
| Glycerol-3-phosphate | 1.093 | 0.12835 | 0.004972 | 2.3035 |
| Guanine | 3.339 | 1.7394 | 3.20E-06 | 5.4952 |
| Guanosine | 0.1108 | -3.174 | 6.96E-05 | 4.1574 |
| Hydrocinnamic acid | 1.9671 | 0.9761 | 0.03192 | 1.4959 |
| Hydroquinone | 1.1424 | 0.19204 | 0.040742 | 1.39 |
| Hypoxanthine | 1.8058 | 0.85261 | 7.24E-05 | 4.1402 |
| Lactic acid | 0.90989 | -0.13623 | 0.0080665 | 2.0933 |
| Leucine | 1.0728 | 0.10141 | 0.0047525 | 2.3231 |
| Maleic acid | 1.1647 | 0.22001 | 0.022246 | 1.6527 |
| Methionine | 1.0983 | 0.13524 | 0.0058974 | 2.2293 |
| Methylmalonic acid | 1.0765 | 0.10636 | 0.014784 | 1.8302 |
| N,N'-Diacetyl-L-cystine-2TMS (putative) | 1.1224 | 0.16661 | 0.031713 | 1.4988 |
| O-acetylserine | 1.0865 | 0.11972 | 0.0040101 | 2.3968 |
| Phenylalanine | 1.0882 | 0.12197 | 0.015067 | 1.822 |
| Proline | 1.1261 | 0.17137 | 0.0026746 | 2.5727 |
| Pyroglutamic acid | 1.0764 | 0.10615 | 0.0071945 | 2.143 |
| Pyruvic acid | 0.60521 | -0.7245 | 0.030074 | 1.5218 |
| Ribose | 1.3236 | 0.4045 | 0.035371 | 1.4514 |
| Scyllo-inositol | 1.077 | 0.10702 | 0.018121 | 1.7418 |
| Serine | 1.0989 | 0.13609 | 0.0033107 | 2.4801 |
| Succinic acid | 1.0835 | 0.11567 | 0.017706 | 1.7519 |
| Threonine | 1.1026 | 0.14096 | 0.0006844 | 3.1647 |
| Thymine | 1.4236 | 0.50953 | 0.01747 | 1.7577 |
| Trehalose | 1.0583 | 0.081711 | 0.010815 | 1.966 |
| Uracil | 2.3353 | 1.2236 | 0.0001762 | 3.7538 |
| Uridine | 0.31982 | -1.6447 | 0.0018232 | 2.7392 |
| Valine | 1.0653 | 0.091258 | 0.0058646 | 2.2318 |
| Xylulose | 1.8033 | 0.85066 | 0.0073295 | 2.1349 |

| **Table S8. Supernatant components in metabolomic footprinting studies of PG45 with significant fold changes (FC) in abundance at 60 min compared to 0 min.** | | | | |
| --- | --- | --- | --- | --- |
| **Compounds** | **FC** | **log_2_(FC)** | **Raw *p* value** | **-log_10_(*p*)** |
| 2-deoxy-D-ribose | 8.6099 | 3.106 | 1.73E-06 | 5.7613 |
| 2-hydroxy-3-methylbutyric acid | 1.1397 | 0.1886 | 0.0063678 | 2.196 |
| 2-hydroxybutyric acid | 0.82591 | -0.27595 | 0.0025664 | 2.5907 |
| 3-hydroxyisovaleric acid | 1.137 | 0.18524 | 0.04046 | 1.393 |
| 3-phenyllactic acid | 1.8286 | 0.87073 | 0.00019317 | 3.7141 |
| 4-hydroxyproline | 1.1712 | 0.22799 | 0.0010998 | 2.9587 |
| Adenine | 2.8409 | 1.5063 | 2.38E-06 | 5.6231 |
| Adenosine | 0.21604 | -2.2106 | 3.29E-06 | 5.4823 |
| Alanine | 1.0742 | 0.10327 | 0.0097459 | 2.0112 |
| Aspartic acid | 1.0743 | 0.10344 | 0.017494 | 1.7571 |
| Cholesterol | 0.68877 | -0.5379 | 0.035049 | 1.4553 |
| Citramalic acid | 1.4443 | 0.53033 | 0.00091101 | 3.0405 |
| Cytosine | 0.63997 | -0.64393 | 0.018264 | 1.7384 |
| Dihydroxyacetone phosphate | 1.1109 | 0.15179 | 0.024628 | 1.6086 |
| Erythritol | 1.265 | 0.33917 | 0.049703 | 1.3036 |
| Glutamic acid | 1.0866 | 0.11976 | 0.015928 | 1.7978 |
| Glutathione | 1.1498 | 0.20143 | 0.030203 | 1.5199 |
| Glyceric acid | 1.124 | 0.16862 | 0.012173 | 1.9146 |
| Glycerol | 1.1266 | 0.17203 | 0.025442 | 1.5944 |
| Glycyl-proline | 1.0814 | 0.11295 | 0.043622 | 1.3603 |
| Guanine | 3.8671 | 1.9513 | 3.25E-06 | 5.4885 |
| Guanosine | 0.093065 | -3.4256 | 8.79E-05 | 4.0559 |
| Hydroquinone | 1.2372 | 0.30713 | 0.033179 | 1.4791 |
| Hypoxanthine | 1.874 | 0.90609 | 0.00012417 | 3.906 |
| Isoleucine | 1.0839 | 0.11628 | 0.024723 | 1.6069 |
| Lactic acid | 0.8494 | -0.23548 | 0.0052211 | 2.2822 |
| Leucine | 1.0856 | 0.11849 | 0.0034218 | 2.4658 |
| Maleic acid | 1.1556 | 0.20868 | 0.023817 | 1.6231 |
| Malic acid | 1.0748 | 0.10409 | 0.045839 | 1.3388 |
| Mesaconic acid | 1.3271 | 0.40825 | 0.043106 | 1.3655 |
| Methionine | 1.0792 | 0.1099 | 0.034188 | 1.4661 |
| Methylmalonic acid | 1.0797 | 0.11063 | 0.0045983 | 2.3374 |
| N-acetyl-L-alanine | 1.0738 | 0.10268 | 0.012234 | 1.9124 |
| Nicotinic acid | 1.0571 | 0.080155 | 0.046674 | 1.3309 |
| O-acetylserine | 1.1083 | 0.14839 | 0.0023252 | 2.6335 |
| Oxoglutaric acid | 0.76434 | -0.38772 | 0.037421 | 1.4269 |
| Phenylalanine | 1.0805 | 0.11174 | 0.0089392 | 2.0487 |
| Proline | 1.1465 | 0.19729 | 0.0037264 | 2.4287 |
| Pyroglutamic acid | 1.0747 | 0.10394 | 0.019726 | 1.705 |
| Pyruvic acid | 0.50004 | -0.99987 | 0.010968 | 1.9599 |
| Ribose | 1.4603 | 0.54625 | 0.0092469 | 2.034 |
| Serine | 1.1016 | 0.13966 | 0.019432 | 1.7115 |
| Succinic acid | 1.072 | 0.1003 | 0.011952 | 1.9226 |
| Threonine | 1.0899 | 0.12414 | 0.0050651 | 2.2954 |
| Thymine | 1.586 | 0.6654 | 0.0065632 | 2.1829 |
| Trehalose | 1.0532 | 0.074819 | 0.048471 | 1.3145 |
| Uracil | 3.1812 | 1.6696 | 4.79E-05 | 4.3193 |
| Uridine | 0.33362 | -1.5837 | 0.0037792 | 2.4226 |
| Valine | 1.084 | 0.11635 | 0.0047601 | 2.3224 |
| Xylulose | 1.9135 | 0.93622 | 0.0045339 | 2.3435 |
